# Antibiotic use among 8-month-old children in Malmö, Sweden – in relation to child characteristics and parental sociodemographic, psychosocial and lifestyle factors

**DOI:** 10.1186/1471-2431-9-31

**Published:** 2009-05-08

**Authors:** Elisabeth Mangrio, Anna Wremp, Mahnaz Moghaddassi, Juan Merlo, Ann-Cathrine Bramhagen, Maria Rosvall

**Affiliations:** 1 Department of Clinical Sciences, Social Epidemiology, Malmö University Hospital, Lund University, Malmö, Sweden; 2 Faculty of Health and Society, Department of Nursing, Malmö, Malmö University, Sweden; 3 Department of Clinical Sciences, Social medicine and Global health, Malmö University Hospital, Lund University, Malmö, Sweden

## Abstract

**Background:**

In the county of Scania, Sweden, antibiotic use among small children is among the highest in the country. The aim of this study was to investigate the associations between antibiotic use among 8-month-old children in Malmö and characteristics of the child as well as parental sociodemographic characteristics, lifestyle factors, and psychosocial support.

**Methods:**

The study was a population-based cross-sectional survey. The study population consisted of children who visited the Child Health Care (CHC) centres in Malmö for their 8-month health checkup during 2003–2006 and whose parents answered a self-administered questionnaire (n = 7266 children). The questionnaire was distributed to parents of children registered with the CHC and invited for an 8-month checkup during the study period.

**Results:**

The odds of using antibiotics increased as parental educational level decreased. Using high educational level as a reference group, low maternal educational level was associated with an increased antibiotic use for the child, odds ratio (OR) = 1.61 (95% CI: 1.34–1.93). Furthermore, children whose parents were born outside Sweden showed higher antibiotic use, OR = 1.43 (95% CI: 1.24–1.65), in comparison with children whose parents were born in Sweden. Exposure to environmental smoking, parental experience of economic stress, and a low level of emotional support increased the odds for antibiotic use. Boys had higher odds of use of antibiotics than girls, OR = 1.40 (95% CI: 1.25–1.57). Having a low birth weight, having an allergy and having siblings also increased the odds for early antibiotic use, while breastfeeding seemed to have a protective role.

**Conclusion:**

There were clear associations between parental factors such as sociodemographic, psychosocial and lifestyle factors and antibiotic use at this early stage of life. Several characteristics of the child also affected the use of antibiotics.

## Background

A survey of 25 European countries in 2003 revealed that Greece and France had the highest out-patient use of antibiotics, while Sweden had a relatively low use compared to the other countries [1]. In addition, antibiotic prescription for children in Sweden decreased by 50% between the beginning of 1990 and the year 2006 [2]. However, antibiotic use within the country is not uniform. One of the highest prevalences of antibiotic use among children in Sweden is found in the county of Scania, in the southern part of Sweden [1,3].

Since there is a correlation between high antibiotic use and the development of resistance [4,5]; a reduction of the relatively high rate of antibiotic use in Scania is of importance; and from a preventive perspective it is important to characterise the children consuming antibiotics. An earlier study from Malmö, Sweden, on 4-year old children, showed that exposure to environmental smoking, attending day care centers, employment of parents and having parents born in Sweden were statistically significant risk factors for using antibiotics [6]. Other studies have shown that the use of antibiotics in children is associated with parental sociodemographic factors such as ethnicity [7], educational level [8-10], families experiencing stress [10], as well as lifestyle factors including parental smoking and exposure to environmental smoking [11]. Certain sociodemographic characteristics of the child, such as being a boy [10,12] or having siblings [13,14], also seem to increase the use of antibiotics. Finally, breastfeeding for more than three [15] or four months [11] has been shown to play an important role in avoiding recurrent antibiotic treatment.

The aim of this study was to investigate the associations between antibiotic use among 8-month-old children in Malmö, Sweden, and characteristics of the child as well as parental sociodemographic characteristics, lifestyle factors, and psychosocial support.

## Methods

### Study population

This study was conducted in Malmö, the third largest city in Sweden. According to statistics provided on the Malmö city website [16], the city has 280801 inhabitants. It is a multiethnic city, with 171 different countries being represented, 27% of the inhabitants being of foreign origin, and every other child born having at least one parent born in another country.

The study was a population-based cross-sectional survey. The study population consisted of children who visited the Child Health Care (CHC) centres in Malmö for their 8-month checkup during 2003–2006 and whose parents answered a self-administered questionnaire (n = 7266 children). The CHC centres in Sweden are a well-established organisation, with responsibility for reducing mortality, morbidity, and disability in newborn and younger children. Another purpose is to educate parents so they can make the most of their child's developmental opportunities. Through regular visits to the child health nurses, each child's weight and physical and developmental health are closely followed, and vaccinations are given until the child is 5–6 years old. The CHC focus is prevention; visits are voluntary and the consultations are free of charge. As many as 99% of children aged 0–6 participate in the programme [17].

The data on the children in the present study was derived from a self-administered questionnaire distributed to parents of children who were registered with the CHC and invited for an 8-month checkup during the study period. The questionnaire was distributed by the pediatric nurses working at the CHC centres.

The questionnaire contained approximately 30 questions about the child's family situation, as well as the parents' education, occupation, country of birth, and financial and emotional security. It also included questions about exposure to environmental smoking, allergies, breastfeeding, and antibiotic consumption. The questionnaire was validated and tested for reliability, and translated into five different languages: Albanian, Arabic, English, Serbo-Croatian, and Somali [18]. In addition to the information provided by the parents, information was also collected from the CHC journal and filled in by the pediatric nurse during the visit.

### Use of antibiotics

*Antibiotic use *was assessed by the question: "Has the child been treated with any form of antibiotics during the child's first eight months of life". The parent could answer yes or no, and also fill in the number of times.

### Parental and child characteristics

*Parents' country of birth *was divided into: both parents born in Sweden, one parent born in Sweden, and both parents born outside Sweden. *Maternal and paternal educational level *was based on years of schooling and divided into 9 years and less, 10–12 years, and more than 12 years of education. *Employment status *was classified into: parental leave (being home with the child), working, and other (unemployed, studying, retirement, or on sick leave). *Maternal smoking during pregnancy *was divided into yes and no. *Exposure to environmental smoking *was divided into no (no exposure at all) and yes (daily exposure, including smoking outside).*Economic stress *was assessed with the question "How many times during the past year did you not have money enough to afford the food or the clothes you and your family need?", with answers being classified into yes (every month, or 6 months a year) and no (very occasionally or never). *Emotional support *was assessed with the question "Do you have someone that can give you proper personal support to cope with life's stress and problems?", with answers being classified into high emotional support (definitely yes or probably yes) and low emotional support (not for certain or no).

Characteristics of the child were also assessed. *Gender *was divided into boy or girl. *Low birth weight *was classified as < 2500 g, and *normal birth weight *as ≥ 2500 g. *Position among siblings in the family *was dichotomised into firstborn versus secondborn or later. *Breastfeeding *was classified into the following categories: 0 months, 0.1–3 months, 3.1–6 months, and 6.1–8 months of breastfeeding. *Still being breastfed at 8 months *was classified as yes or no. *Allergies *were categorised by the answer to the question "Does the child have allergies?" to which the parent could answer yes or no.

### Statistical methods

Odds ratios (OR) and 95% confidence intervals (95% CI) were used to analyse the associations between antibiotic use and the various child and parental characteristics. Multiple logistic regression analyses were performed in order to adjust the estimated OR for the influence of confounding factors. Model 1 included year and gender; model 2 included year, gender, and parents' country of birth; model 3 included year, gender, parents' country of birth, and maternal educational level; and model 4 included year, gender, parents' country of birth, maternal educational level, and having had recurrent infections. Statistical analyses were performed with version 12.0.1 of SPSS for Windows. The study was approved by the Regional Ethical committee, Lund University.

## Results

The study included 7266 8-month-old children over the four years of the study period (2003–2006). Table 1 shows the number of children and prevalence of antibiotic use for each year.

**Table 1:** Prevalence of antibiotic use and total number of 8-month old children participating per study year.

Year	Antibiotic use n (%)	Total number of children N
2003	437 (25.0)	1806
2004	422 (22.9)	1904
2005	317 (19.8)	1664
2006	374 (21.3)	1892

Table 2 describes the associations between parental sociodemographic characteristics, lifestyles, and psychosocial factors and antibiotic use among children at the age of 8 months. Antibiotic use showed no statistically significant associations with parental age (data not shown), paternal employment status (data not shown), maternal employment status, or maternal smoking during pregnancy. The crude odds for early antibiotic use were significantly higher in families where both parents were born outside Sweden, OR = 1.43 (95% CI: 1.24–1.65), compared to families where both parents were born in Sweden. The odds remained statistically significant even after adjustment for potential confounders. Using high educational level (> 12 years of education) as a reference group, low maternal educational level (≤ 9 years of education) was associated with an increased antibiotic use for the child, OR = 1.61 (95% CI: 1.34–1.93). A similar pattern of association was seen for paternal educational level (data not shown). This association was only slightly reduced after adjustment for potential confounders, but turned statistically non-significant in the last model. Daily exposure to environmental smoking was associated with an increased antibiotic use. This association remained after adjustment for year, gender, and parents' country of birth, but decreased after adjustment for maternal educational level and recurrent infections. Families experiencing economic stress and reporting low access to emotional support had a significantly increased use of antibiotics among their 8-month-old children, OR = 1.55 (95% CI: 1.24–1.93) and OR = 1.47 (95% CI: 1.26–1.71), respectively. These associations remained statistically significant after adjustment for potential confounders.

**Table 2:** Associations between parental sociodemographic characteristics, lifestyle and psychosocial factors and antibiotic use in 8-month old children, Malmö, Sweden.

Characteristics	Use of antibiotics n (%)	Crude OR 95% CI	Adjusted OR 95% CI Model 1^a)^	Adjusted OR 95% CI Model 2^b)^	Adjusted OR 95% CI Model 3^c)^	Adjusted OR 95% CI Model 4^d)^
**Parents' country of birth**						
Parents born in Sweden	837 (20.8)	1.00	1.00		1.00	1.00
One parent born in Sweden	254 (20.9)	1.00 (0.85–1.17)	1.01 (0.86–1.19)		0.98 (0.83–1.15)	1.09 (0.91–1.32)
Parents born outside Sweden	378 (27.4)	1.43 (1.24–1.65)	1.47 (1.28–1.70)		1.35 (1.16–1.57)	1.73 (1.44–2.07)
**Maternal educational level**						
> 12 years	800 (20.1)	1.00	1.00	1.00		1.00
10–12 years	512 (24.2)	1.27 (1.12–1.44)	1.28 (1.12–1.45)	1.22 (1.07–1.40)		1.15 (0.99–1.34)
≤ 9 years	202 (28.9)	1.61 (1.34–1.93)	1.67 (1.39–2.01)	1.47 (1.19–1.80)		1.25 (0.98–1.60)
**Maternal employment status**						
Parental leave	1254 (22.2)	1.00	1.00	1.00	1.00	1.00
Working	117 (19.8)	0.86 (0.69–1.06)	0.88 (0.71–1.09)	0.90 (0.73–1.12)	0.91 (0.73–1.13)	1.01 (0.78–1.30)
Other	141 (24.2)	1.11 (0.91–1.36)	1.09 (0.89–1.33)	1.00 (0.81–1.24)	0.99 (0.80–1.23)	0.98 (0.76–1.26)
**Maternal smoking during pregnancy**						
No	1353 (22.0)	1.00	1.00	1.00	1.00	1.00
Yes	138 (25.1)	1.19 (0.97–1.46)	1.20 (0.97–1.47)	1.12 (0.91–1.39)	1.02 (0.81–1.27)	0.93 (0.72–1.20)
**Environmental smoking**						
No	1207 (21.4)	1.00	1.00	1.00	1.00	1.00
Yes	313 (25.5)	1.25 (1.09–1.45)	1.27 (1.10–1.47)	1.20 (1.03–1.40)	1.10 (0.94–1.29)	0.97 (0.80–1.17)
**Economic stress**						
No	1334 (21.6)	1.00	1.00	1.00	1.00	1.00
Yes	124 (30.0)	1.55 (1.24–1.93)	1.58 (1.27–1.98)	1.47 (1.16–1.86)	1.38 (1.08–1.75)	1.44 (1.09–1.91)
**Emotional support**						
High	1239 (21.3)	1.00	1.00	1.00	1.00	1.00
Low	266 (28.4)	1.47 (1.26–1.71)	1.49 (1.27–1.74)	1.35 (1.13–1.61)	1.29 (1.07–1.54)	1.40 (1.13–1.73)

^a) ^Adjusted for year and gender

^b) ^Adjusted for year, gender and parents' country of birth

^c) ^Adjusted for year, gender, parents' country of birth and maternal educational level

^d) ^Adjusted for year, gender, parents' country of birth, maternal educational level and recurrent infections

Table 3 shows the association between antibiotic use and characteristics of the child such as gender, birth weight, position among siblings in the family, breastfeeding, and having an allergy. Significantly more boys than girls had been treated with antibiotics at 8 months of age, while children with a low birth weight had nearly 50% increased odds for having used antibiotics. These associations were only slightly reduced after adjustment for potential confounders. Having siblings was also associated with increased odds of using antibiotics, and this association remained after adjustment for confounders. Most of the children were breastfed for between 3.1 and 6 months. Using this group as the reference, children who were never breastfed at all had significantly increased odds of using antibiotics, OR = 1.71 (95% CI: 1.26–2.31). This result was only slightly changed after adjustment for potential confounders. Children with an allergy had increased odds for early antibiotic use, OR = 1.65 (95% CI: 1.20–2.27). This association was only slightly reduced after adjustment for potential confounders, but turned statistically non-significant in the last model.

**Table 3:** Associations between child characteristics and antibiotic use in 8-month old children, Malmö, Sweden.

Characteristics	Use of antibiotics n (%)	Crude OR 95% CI	Adjusted OR 95% CI Model 1^a)^	Adjusted OR 95% CI Model 2^b)^	Adjusted OR 95% CI Model 3^c)^	Adjusted OR 95% CI Model 4^d)^
**Gender**						
Girl	645 (19.2)	1.00	1.00	1.00	1.00	1.00
Boy	862 (25.0)	1.40 (1.25–1.57)	1.39 (1.24–1.57)	1.42 (1.26–1.60)	1.44 (1.28–1.62)	1.35 (1.18–1.55)
**Low birth weight** **(< 2500 g)**						
No	1453 (22.0)	1.00	1.00	1.00	1.00	1.00
Yes	85 (28.7)	1.43 (1.10–1.85)	1.48 (1.14–1.93)	1.50 (1.15–1.96)	1.49 (1.13–1.96)	1.87 (1.36–2.58)
**Nr in the family of brothers and sisters**						
Firstborn	592 (16.9)	1.00	1.00	1.00	1.00	1.00
Secondborn and more	831 (27.5)	1.86 (1.65–2.09)	1.88 (1.67–2.12)	1.89 (1.67–2.14)	1.86 (1.64–2.11)	1.39 (1.20–1.60)
**Breastfeeding**						
0 months	71 (32.1)	1.71 (1.26–2.31)	1.75 (1.28–2.38)	1.75 (1.28–2.40)	1.64 (1.19–2.26)	1.92 (1.31–2.80)
0.1–3 months	211 (24.8)	1.19 (0.98–1.43)	1.18 (0.97–1.43)	1.11 (0.91–1.36)	1.03 (0.84–1.27)	0.97 (0.77–1.24)
3.1–6 months	430 (21.7)	1.00	1.00	1.00	1.00	1.00
6.1–8 months	72 (23.2)	1.09 (0.82–1.45)	1.11 (0.83–1.48)	1.01 (0.75–1.37)	0.99 (0.73–1.34)	0.92 (0.65–1.32)
**Still breastfeeding at the age of 8 months**						
Yes	712 (21.2)	1.00	1.00	1.00	1.00	1.00
No	718 (23.2)	1.12 (0.99–1.26)	1.11 (0.99–1.25)	1.14 (1.01–1.29)	1.10 (0.97–1.25)	1.09 (0.94–1.26)
**Allergies**						
No	587 (20.0)	1.00	1.00	1.00	1.00	1.00
Yes	59 (29.2)	1.65 (1.20–2.27)	1.66 (1.20–2.28)	1.56 (1.12–2.19)	1.62 (1.16–2.28)	1.40 (0.93–2.10)

^a) ^Adjusted for year and gender

^b) ^Adjusted for year, gender and parents' country of birth

^c) ^Adjusted for year, gender, parents' country of birth and maternal educational level

^d) ^Adjusted for year, gender, parents' country of birth, maternal educational level and recurrent infections

## Discussion

This population-based study of 8-month-old children in the city of Malmö, Sweden, showed that the use of antibiotics up to this young age was increased among children whose parents were born outside of Sweden, had a low educational level, experienced economic stress, or had low emotional support. Although exposure to environmental smoking was associated with an increased antibiotic use in the crude model, this association was attenuated and turned non-significant after adjustment for potential confounders. Breastfeeding seemed to have a protective role against antibiotic treatment. Furthermore, the odds of antibiotic use were significantly increased by being a boy, having a low birth weight, having siblings, and having an allergy.

In our study, antibiotic consumption was significantly higher among children whose parents were less well educated. Similar results have been shown in other studies [8,10]. The reasons for this result might include an increased exposure to infectious agents due to crowding, poor nutrition, smoking, and stress [10]. However, there are also studies that have shown a lower use of antibiotics in families where the parents had a low educational level [9]. Use of antibiotics was also higher in families where both parents were born outside of Sweden, a finding supported by another study performed in the USA [7]. This could be partly due to different cultural traditions in relation to having a disease. Earlier studies, including a study from Malmö, Sweden, among 4-year old children, have shown an association between exposure to environmental smoking and increased antibiotic use [6,11]. We found a similar pattern of association in the crude model of our study, but the association turned non-significant after adjustment for potential confounders. Earlier studies have shown a higher antibiotic consumption among the children of families experiencing stress and parents in need of support from outside the family [10,12]. Our study showed similar results, with higher antibiotic use among parents with low emotional support as well as among parents experiencing economic stress. As in other studies [10,12], boys were prescribed antibiotics more frequently. Furthermore, children who were not breastfed at all had increased odds of using antibiotics. This finding of a protective role from breastfeeding is supported by other studies [11,15].

Certain methodological issues need to be addressed. All parents with 8-month-old children were invited to participate in the study by answering the questionnaire. The total number of children whose parents answered the questionnaire during the four-year study period was 7266, or about two thirds of all those who received the questionnaire. We have no information about the country of birth in the non-participating families, but according to Rosvall et al. [18], the non-participants had a somewhat higher proportion of parents born outside of Sweden. This might have resulted in an underestimation of the total prevalence of antibiotic consumption, since our results indicate that there was a higher antibiotic consumption among children with both parents born outside of Sweden. There were no educational differences between the participants and non-participants [18]. One strength of this study was the fact that the questionnaire had been validated and tested for reliability and translated into five different languages [18]. According to the Malmö city website, these languages are among the most common languages represented in Malmö [16]. However, there may still have been some bias, as despite the multiple translations some parents may have had difficulties in understanding the questions. Another problem with the questionnaire was that some questions may be more sensitive than others (smoking, economy, and emotional support), and some parents may not have been willing to answer them. We chose to adjust for the following confounding factors: male gender, parents' country of birth, parental educational level, and recurrent infections. Since there were differences in antibiotic consumption during the different years of the study, we also adjusted for the year. However, there might have been other confounding factors not included in our models.

## Conclusion

Our results showed that the use of antibiotics among 8-month-old children in Malmö, Sweden, was influenced by several factors including parental sociodemographic factors, lifestyle factors, psychosocial support, as well as child-related factors. The parental characteristics associated with higher antibiotic use were low educational level, being born outside Sweden, economic stress, and having low emotional support. The child-related characteristics associated with higher antibiotic use were male gender, low birth weight, having siblings, having an allergy, and not being breastfed at all.

## Competing interests

The authors declare that they have no competing interests.

## Authors' contributions

EM, AW and MR have contributed to the conception of the work, the analysis of the data, the interpretation and the discussion of the results, the drafting, writing, and revision of the content. JM and A-CB have contributed to the interpretation and the discussion of the results. All authors read and approved the final manuscript. MM has contributed with help of the statistical work behind this article.

## Pre-publication history

The pre-publication history for this paper can be accessed here:


